# Abstract of Closing Remarks in His Address before the Association

**Published:** 1891-01

**Authors:** V. A. Howeth

**Affiliations:** Retiring President of the North Texas Medical Society


For Daniel's Texas Medical Journal.

### ABSTRACT OF CLOSING REMARKS IN HIS ADDRESS BEFORE THE ASSOCIATION.

BY V. A. HOWETH, M. D.,
Retiring President of the North Texas Medical Society.

I HAVE already, for this occasion, said enough, but, with your permission and indulgence, I will embrace the opportunity to present a few matters I hope of interest and practical importance deserving the especial attention of medical men, and to a greater or less extent the public also.

Upon the occasion of our last meeting in your city, we were honored with a masterly and scholarly address by one of Texas' ablest citizens, honored statesman, and, above all, an honest man, the Honorable T. J. Brown, of your city.

His discourse was a most forcible and pointed contrast or parallelism between the legal and medical professions. The speaker did us the compliment to argue and maintain that the two professions were fully equal in point of usefulness and high and honorable standing, and proceeded to explain the simple but rigid and inflexible rules, or system of ethics, whereby the legal profession was enabled, by its own inherent regulating power, to protect itself, and the public, in the main, from legal frauds and disreputable pretenders or shysters, while the very reverse was true concerning the medical profession; even the most illiterate and unworthy, by public acquiescence and sanction, being allowed to roam the country at will and ply their infamous arts and deceptions of the most unblushing, vile and degrading quackery, this being permitted by law and sanctioned by an unwitting or perverted public sentiment.

The honorable speaker seriously grappled with the subject, and gave us the benefit of his advice, and encouraged us to hope for better things.

It is a deplorable fact that the medical profession, alone and unaided, is unequal to the work of reform in this matter, and it seems that under no stress of circumstances, for the purity of the profession and the protection of the people, can we successfully call to our aid the united moral support of the people, nor a legislative helping hand. And notwithstanding all our struggles and efforts to successfully maintain the banner of pure scientific medicine and transmit it untarnished to those who must follow us, the medical profession itself stands indicted by the world, for the silly quackery and death dealing charlatanry practiced in its name.

The time was when the title, doctor, carried with it the idea of some degree of learning, respectability, decency and dignity. In fact, the title was one of honor, and universally so acknowledged. But now any ruffian is called doctor who masquerades our streets with long dishevelled hair and outlandish garb, and amid ravings, vulgarity and humorous or indecent songs plies his villainous business of speculating upon the ignorance and gullibility of the people. Yes, he is titled doctor, reinforced by the adjectives, distinguished, celebrated, renowned, etc.

What a travesty upon respectability, common decency, an honorable profession and its honored men of refinement, culture, and scientific attainments.

What disgrace, shame and degradation such contrast brings to view. This infamy is so plain that the public must be able to see it, even at a glance, and may we not hope that the public will, more and more, come to our assistance in maintaining and fostering a pure and honest system of medicine, that will be an honor and a blessing to our children and to future generations.

. The libel law of Texas is such that neither the people, the medical journals nor the general press dare attempt to expose even the most notorious, villainous charlatanry. If they do, a heavy risk is incurred. The law should be corrected so as to promote the ends of justice, rather than defeat them.

Another thing worthy our serious consideration as medical men. As far as my knowledge extends, it seems a fact that our profession is unjustly discriminated against in all matters of public service of whatsoever character. For instance, every well regulated city has its government, and it must have a city attorney and a city physician. And when we come to examine the pay-roll, we find that, as a rule, the attorney receives a salary at least two to five times greater than the physician, although the duties of the latter are usually equally responsible and more arduous and burdensome to perform. The two professions are admitted alike honorable, useful and indispensable. Then why this distinction without a difference?

Then again, counties must have their county physicians. The commissioners generally advertise for bids for this responsible position, which is practically putting it up at auction, and it usually goes to the lowest bidder. The only difference between this and buying a brute at auction is that the brute always goes to the highest bidder. I have known physicians, in the scramble for this service, to bid a sum per annum wholly insufficient to supply an average line of necessary medicine. It is a crowning shame, a libel upon our civilization, discreditable to our profession, and criminally unjust to the poor and unfortunate who are interested to the full extent of their health and lives, like other human beings.

When we ascend the scale, and get up into higher government circles, where men must be selected to fill responsible positions in appropriate state departments, we find that it is not always the fitness of the man for the place that governs the appointment, but rather the amount and character of political influence brought to bear. These positions should never be subject to, or controlled by the political influences to the extent they are. Incompetent and unworthy men, who may happen to know the moves on the political checker board, stand as good, if not a better, show for appointment than the most worthy and well qualified. Justice and humanity demand that the appointing power should rise above political corruption in these matters, which are truly of great moment, and should be sacredly so regarded.

Furthermore, the state inflicts gross injustice upon physicians by compelling them to serve as experts before the courts in State cases, without compensation, oftentimes forcing them to a distance for an indefinite time, at possibly a heavy personal expense, besides a total loss of time and suspension of business. This imposition is shameful in the extreme. Then again, if in the affairs of the State any exigency arises requiring medical skill and attention, the State usually turns a deaf ear to even modest and reasonable demands for compensation, for whatever service in its behalf. For a most shameful instance of such cruel injustice, read Dr. Johnston's experience, as given in the last number (November, 1890) of DANIEL'S TEXAS MEDICAL JOURNAL. The editor, in commenting upon it, truthfully says, "The highwayman has as much right to despoil the traveler of his personal effects at the muzzle of the six-shooter" as the State has to force physicians to perform important and responsible duties in its behalf without a just and equitable remuneration therefor. It seems that of all trades, callings and professions, medicine alone has no redress at law. Surely there should be a remedy provided for this lamentable state of affairs." Like others of the State's citizens, we are tax-payers, and do everything in our power for the substantial support and welfare of the government. We bear our part in sustaining its institutions and burdens, and are ever ready to give counsel freely in relation to all matters or measures appertaining to public health, sanitation, etc.

I do not charge or regard this as a matter of invidiousness or discrimination against our profession, but rather from the lack of a proper concern or conception of legitimate legislative needs in establishing relations between the government and the medical profession. It is a fact that the legislative powers of the State, from time to time, upon all matters medical, have exhibited the least degree of concern and the greatest amount of folly and stupidity. This is probably owing to the fact that so few physicians, who better understand these needs, take an active part, directly or indirectly, in shaping legislation, this being done principally by those from other business pursuits or callings, notably our friends of the legal profession, who are amply fortified and protected against impositions from whatever source, from the justice's precinct court up to the highest tribunal in the land. There is no such thing as forcing them to do gratuitous work for the State or the public. I do not offer this as a criticism, but to justify and commend their discreetness and sagacity. Their gratuitous labors, like their charitable deeds, are voluntarily bestowed, being neither required nor forced by law, circumstance nor condition. But with us the diversified demands of humanity are inexorable, and whatever burden is presented, we must bear. There is no escape. Under all these circumstances is it not a cruel wrong, a crowning injustice for the State also to lay its oppressive hand so heavily upon us by exacting our time, knowledge and skill without compensation?

Another gigantic imposition and injustice also demands consideration at our hands. As has already been said, there is no profession more noble and philanthropic than the medical. There is none from which greater, or even equal demands for kindly and gratuitous service is required or rendered. Independently of legitimate charities and gratuities, it seems to be a part of the machinery of the universe that the physician, every day of his professional life, shall render some service, at whatever cost of time or talent, without the hope of fee or reward, and regardless even of thanks or of gratitude. In this matter, our profession is called upon to exercise a greater degree of magnanimity and forbearance than is required of all other professions or callings combined. The more we concede, the more it seems is required. The yoke sometimes gets heavy and burdensome, and we become footsore and weary, and forget that there may still be virtue in forbearance, or that out of evil good may come. In thus considering the question, it seems wholly contrary to the natural order of things, when a great corporate money power, as combined in railway systems, should not only ask, but positively demand that the medical profession shall also serve their interests upon a scale of enforced benevolence (which they call business) unequalled in the experience of the profession. Orphan homes and the direst poverty could hardly presume to ask more. Insiduously has concession after concession been demanded of us, and granted, until the iron heel of railway corporations is firmly planted across our necks, professionally speaking. We have surrendered to them at their own bidding, and upon their own terms. The local physician and surgeon, without discretion, has to accept whatever is offered, and the physician and surgeon not in the so-called employ can scarcely, uhder any stress of circumstances, obtain even a pittance in way of remuneration for occasional emergent humane service to meet extreme exigencies. Dr. R. M. Swearingen, in a paper somewhat upon this subject, read before the Austin District Medical Society in 1888, and published in DANIEL'S TEXAS MEDICAL JOURNAL, says, "The old-fashioned scales of equity and justice, used in the ox-cart days, for balancing rights and wrongs, are thrown aside, and the voice of humanity and reason seems lost in the rattle of wheels and the screams of engines, . . . and that when acting in the body corporate, these railway combines attend strictly to financial affairs and look at all things purely from a standpoint of profit and loss," regardless of the just and moral obligations which inevitably exist between man and man. They attend to business with a degree of exclusiveness that turns the brain into a ledger and the heart into stone.

The medical departments of railway systems are indispensable. Humanity demands them, and their responsibility is great. Yet it is the poorest paid of all the departments; and for which we are possibly largely to blame oursilves, because of our humble, quiet submission to a selfish dictation in the matter. The law and business departments of these systems are also indispensable, and involve responsible duties, but they are respectably and even generously supported and paid, which is right and proper. And why should our profession be so ungraciously abased by the mercenary will or dictum of any power that our performance of important and responsible duties is reduced to a mere matter of empty servility?

Although fully conversant with all the pros and cons of the subject, by reason of several years experience in the service, I have never yet heard a reasonable or tenable excuse or apology for this discrimination against the medical profession, reducing, as it does, our services to about a common level with unskilled day labor.

Railway corporations, with all their cunning, are not catalogued or classed as pauper institutions, nor do they successfully pose in the role of organizations in need of charitable or eleemosynary consideration, for besides legitimate earnings and oppressive extortions upon the transportation and commerce of the country, most of them, at least, have been largely enriched by munificent donations of public domain, and unrestrained have laid their hands heavily upon almost every city, town and village, levying and exacting the most unjust and burdensome tribute, in way of prodigious money subsidies, thereby touching the pockets of almost every citizen; and still unsatisfied and insatiable, every advantage and opportunity is utilized of exacting the maximum amount of service for the minimum pay in return, oftentimes grinding and reducing the wage-worker to the most unmerciful and dire straits of poverty.

Dr. Swearingen, whom we have previously quoted, says that out of this principle has largely sprung that "unnamed element of discord between poverty and wealth, between labor and capital," and out of which has emerged the grim monster of modern anarchy and communism, at least it is one of the great factors producing such demoralization.

The doctor also says that "redress, single-handed, being impossible, the strategy of war suggested the combination of forces," and therefore the laboring classes were driven into organized labor unions and brotherhoods for reciprocal safety and mutual protection. The principle seemed to be, that labor was forced to combine in proportion to the combination of capital in these and kindred giant corporations. For money, with all the conveniences and good that adheres to it, as a power of oppression, when so used, is the most heartless and soulless.

The doctor, who is himself a statesman, concludes that "the subject is one worthy the profoundest thought of every patriotic citizen." His words are full of truth and wisdom, for railway managements seem interested more in watered stocks, financial problems, etc., than in orthodoxical questions of ethics or humanity.

Of course we have nothing directly to do with this phase of the subject, but it fairly illustrates the lilliputian spirit or principle in which is grounded their unjust and iniquitous demands upon the medical profession. Unhappily for us, ours has proven a most fertile and fruitful field from which a rich harvest has been and is being gathered by them at our expense, together with that of taxed employees. However, I do not think that railway managements are always as cold-blooded and wicked as many people believe; but one thing is sure, they will take every advantage and every inch of ground, as well as every penny, if we but say nothing and allow them the privilege. It seems they have an aching void which they hope to fill by triumphal conquest, subduing the universe to their own ends and purposes. They concede no individual rights, they respect no man nor class of men except upon the stern basis of law, and there is no advantage they will not take, however unjust or iniquitous, to serve the most sordid and selfish economic pretenses.

And when in the so-called employ, our service is required free of charge, in the capacity of experts or otherwise in any or all the courts, whenever called upon. In fact, we must dance a quickstep to whatever music is wafted to our ears, and are required to pay the fiddler besides. However, subordinate officials with an itching desire to gain favor with superiors, are frequently to blame for unbecoming and unseemly exactions.

The natural tendency and drift of the evils set forth is to depreciate and lower the time-honored standard of professional dignity and usefulness sacredly bequeathed us by our worthy and illustrious forefathers, and degrade more and more toward a common level with cheap, common, clap trap charlatanry.

Let us, like true, valiant soldiers in a righteous cause, make a trench here and throw up embankments there, and under a flag that knows no truce, sheltered behind a defense, doubly fortified by all that is sacred, pure, true and noble, bravely defend our profession against being undermined of its legitimate honors, prosperity and rewards, and being left like a mighty, glittering, towering temple, despoiled by remorseless, ignoble hands, to be trampled upon as a common, crumbled ruin.

We should not be overawed into a compromise, nor barter our heritage of prefessional rights and prestige for a mere mess of pottage, nor allow ourselves whipped into servile subjection to or by any authority, subordinate or supreme.

Let us not be as dumb driven cattle, but in a spirit of fairness be just, and demand equal justice to all concerned, though the heavens fall. And while railway systems are requiring everything of us, let us, like noblemen of a noble ancestry, also require an adequate appreciation and a proportionate just reward or recompense, becoming and befitting the character and dignity of our profession.

In all truth and candor, I feel that we have been too easily beguiled into a Rip Van Winkle sleep upon this matter. We should wake up from this seductive slumber and its delusive dreams. Our professional relations and responsibilities to these systems are something more and superior to that of the liveried Pullman porter, although he treads the palatial sleeper, with its gorgeous furnishings and silvery mountings, with the air of a prince or magnate, happy in the thought of receiving more genteel, or at least a greater proportionate compensation for services rendered than the local physician and surgeon, if not the subordinate chief surgeon himself. Look at it as we may, and think of it as we will, in the light of approved reason and judgment, is it not a burning shame?

Life is real, life is earnest, and the frowning clouds and sombre shadows are, if possible, more potently real than the faultless beauties of azure tints, the starry decked heavens, or the wondrous prismatic coloring of the enchanting rainbow; and while it would delight us more to contemplate and discuss the more pleasing features of our professional life and experience, I thought it not untimely, upon this occasion, to follow in the footsteps of Sam Jones and call attention to some of the wickedness of the times affecting the medical profession. If these matters are never agitated by the profession, when, and by whom will they ever be? In denouncing the wrong and contending for the right, even those whose will and interests are directly opposed will honor manly courage and devotion to principle.

Let us look at this matter in no sordid way, but rather upon honest, wholesome, ethical and business principles, adhering to the strictest professional honor and integrity, ever thinking and acting in conformity to the hallowed teachings of the golden rule, to "do unto others as we would have them do unto us."

I again thank you most heartily for your kindly hospitalities and patient indulgence.

---

Compound tincture of benzoin and glycerine, equal parts, applied to fissured nipples, will seldom fail to cure, if the parts are kept clean.

